# Nek7 is overexpressed in hepatocellular carcinoma and promotes hepatocellular carcinoma cell proliferation *in vitro* and *in vivo*

**DOI:** 10.18632/oncotarget.7620

**Published:** 2016-02-23

**Authors:** Lei Zhou, Zhixin Wang, Xinsen Xu, Yong Wan, Kai Qu, Haining Fan, Qiangpu Chen, Xuejun Sun, Chang Liu

**Affiliations:** ^1^ Department of Hepatobiliary Surgery, The Affiliated Hospital of Binzhou Medical University, Binzhou, China; ^2^ Department of Hepatobiliary Surgery, The First Affiliated Hospital, School of Medicine, Xi'an Jiaotong University, Xi'an, China; ^3^ Department of General Surgery, The First Affiliated Hospital, School of Medicine, Xi'an Jiaotong University, Xi'an, China; ^4^ Department of Hepatopancreatobiliary Surgery, The Affiliated Hospital of Qinghai University, Xining, China

**Keywords:** Nek7, hepatocellular carcinoma, cell proliferation, cyclin B1, prognosis

## Abstract

NIMA-related kinase-7 (Nek7) is a serine/threonine kinase involved in cell-cycle progression via mitotic spindle formation and cytokinesis. In this study, we investigated whether Nek7 involves in hepatocellular carcinoma (HCC). Interestingly, we found that Nek7 was significantly overexpressed in HCC than in liver tissues. In HCC patients, high Nek7 expression was significantly correlated with tumor numbers, tumor diameter, adjacent organs invasion, tumor grade and TNM stage. Furthermore, Nek7 expression pattern showed close relationship with that of Ki-67, a well-stablished cell proliferation marker. More importantly, patients with higher expression levels of Nek7 had significantly lower 5-years survival rate. Likewise, Nek7 expression was significantly higher in HCC cell lines than normal hepatic cell line. By Nek7 silencing using lentivirus-mediated Nek7 interference approach, the growth of HCC cell lines was inhibited and the tumor growth in xenograft mouse model was also suppressed. Mechanistic studies showed that silencing of Nek7 resulted in decreasing cyclinB1 level both *in vitro* and *in vivo*. In conclusion, this study highlights for the first time the possible role of Nek7 in HCC progression. Nek7 would be a useful biomarker that early predicts HCC patients at higher risk of poor prognosis. Also, Nek7 could be a novel HCC therapeutic target.

## INTRODUCTION

Hepatocellular carcinoma (HCC) is the fifth most common malignant tumor, and the second most common cause of cancer deaths worldwide [1-2]. In spite of the improvements in HCC early diagnosis and treatment, the 5-year survival rate remains unsatisfactory [3]. Many studies have investigated the HCC progression, tumorigenesis and signaling pathways suggesting new insights for HCC therapy [4]. Therefore, identification of target molecules and molecular mechanisms underlying the development and progression of HCC are of particular importance.

In this context, The Nek (NIMA Related Kinase) protein kinase family includes 11 members which were originally identified as human orthologs to the Aspergillus nidulans protein kinase, NIMA (Never In Mitosis, gene A) kinase [5-6]. NIMA is involved in regulating cell cycle and mitotic progression [7-10]. In particular, Nek7 is involved in spindle formation during mitosis and activation of the spindle assembly checkpoint [11]. Moreover, Nek7 is highly expressed in gallbladder cancer compared to normal tissues, and has significant relationship with tumor differentiation, metastasis and patients survival rate [12]. On the other hand, Ki-67 is a well-established biomarker for cell proliferation and its biological role in the HCC progression has been suggested. [14-18].

In this study, we showed, for the first time, the differential expression pattern of Nek7 in HCC tissues. In addition, we examined the correlation between Nek7 expression pattern and clinico-pathological features of HCC patients and survival rate. Also, we found a close relationship between Nek7 and Ki-67 indicating that Nek7 might play an important role in HCC cell proliferation. To address this, we used lentivirus-mediated specific shRNA targeting Nek7 to investigate the impact of Nek7 silencing on the HCC cell proliferation and SMMC7721 xenograft tumor growth. This approach provided evidence that Nek7 promotes HCC cell proliferation via regulating cyclin B1. Taken together, this study proposes Nek7 as a novel tumor associated gene.

## RESULTS

### Nek7 expression in HCC tissues and HCC cell lines

**Figure 1:**
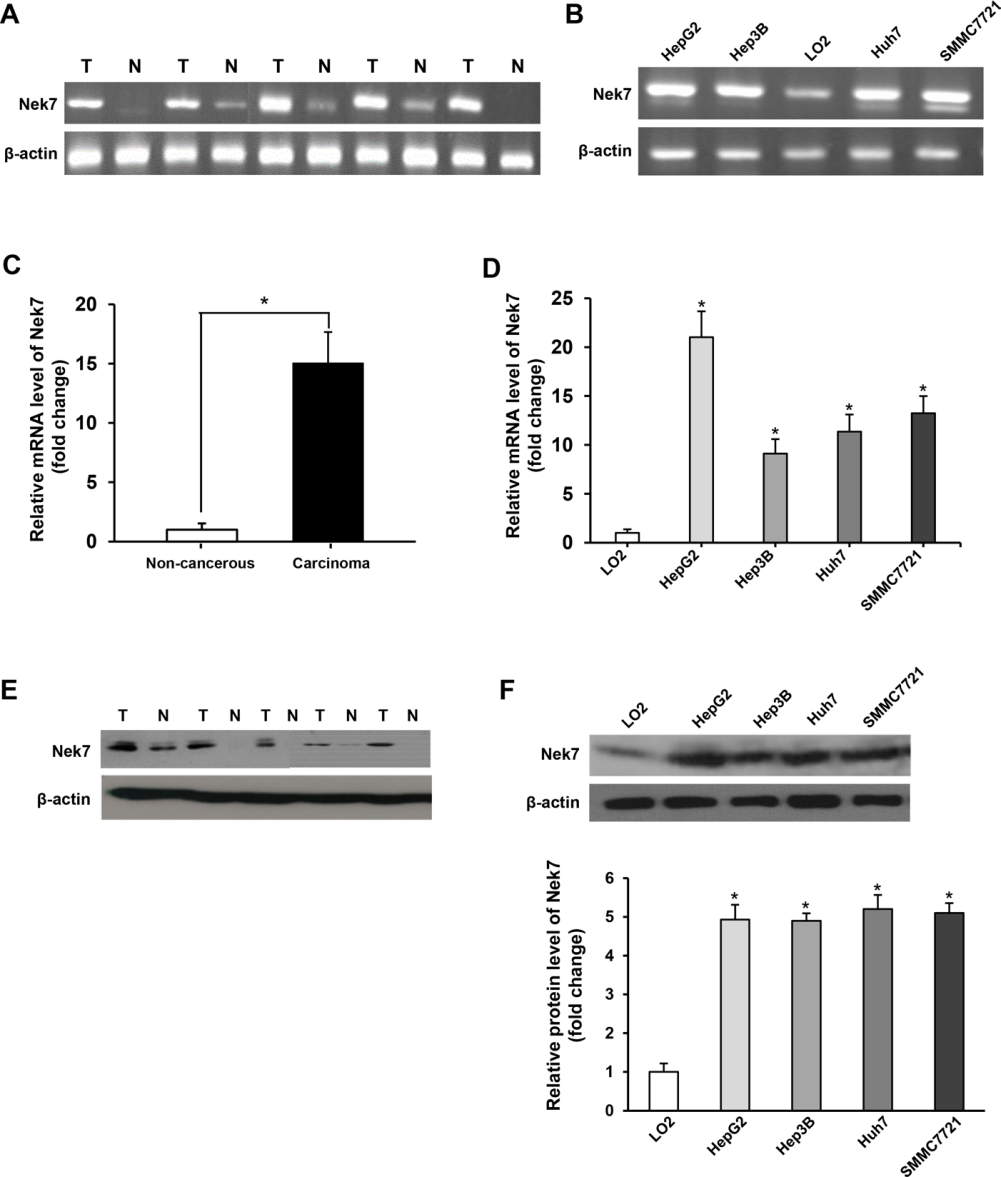
The results of Nek7 mRNA and protein expression in tissue specimens and cell lines **A.** Semi-quantitative RT-PCR analysis of Nek7 mRNA expression in HCC specimens (T) and normal liver tissues (N). **B.** Semi-quantitative RT-PCR analysis of Nek7 mRNA expression in HCC cell lines (HepG2, HuH7, Hep3B and SMMC7721) and a normal hepatic cell line (LO2). **C.** Real-time PCR analysis of Nek7 expression in HCC specimens (T) and normal liver tissues (N). **D.** Real-time PCR analysis of Nek7 expression in HCC cell lines and normal hepatic cell line LO2. **E.** Western blot analysis of Nek7 protein expression in representative HCC (T) and normal liver tissues (N). **F.** Western blot analysis of Nek7 protein expression in four HCC cell lines and a normal hepatic cell line (LO2). β-actin was used as internal control for RT-PCR and Western blot. Experiments were done in triplicate. (*p < 0.05).

First we used a semi-quantitative RT-PCR assay to examine the expression level of Nek7 in HCC. This clearly demonstrated, that Nek7 was significantly upregulated in HCC specimens compared to the normal liver samples (Figure 1A). In support of these results, Nek7 expression was also significantly higher in the HCC cell lines (HepG2, HuH7, Hep3B and SMMC7721) than in the normal hepatic cell line; LO2 (Figure 1B). To further confirm this finding, the mRNA level of Nek7 was determined by quantitative RT-PCR analysis in 80 HCC tissues and their matched non-cancerous liver tissues. The mean expression of Nek7 in HCC tissues was approximately 15-fold higher compared to that of the corresponding normal tissues (Figure 1C). Furthermore, the Nek7 expression was approximately 22, 9, 11 and 13-fold higher in HepG2, Hep3B, Huh7 and SMMC7721, respectively, than that of normal liver cell line LO2 (Figure 1D). We also examined the expression level of Nek7 protein in HCC and normal liver tissues and liver cell lines. This analysis revealed that Nek7 protein was overexpressed in HCC specimens but not in the matched normal adjacent liver tissues (Figure 1E). Similarly, the Nek7 protein level was higher in HCC cell lines than normal cell line; LO2 (Figure 1F). Taken together, these results suggested that Nek7 involves in HCC.

### Analysis of Nek7 expression by immunohistochemistry

**Figure 2:**
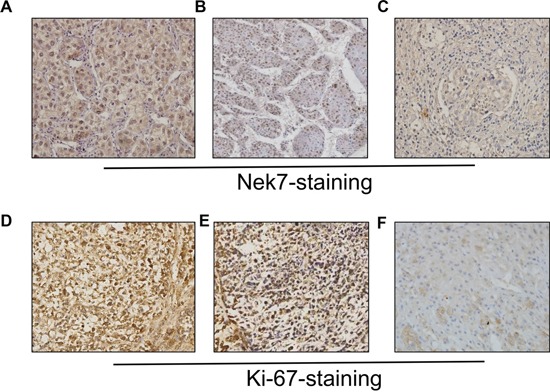
Immunohistochemistry of Nek7 and Ki-67 on HCC specimens Representative immunohistochemical staining of HCC specimens with different histologic grade, as determined using anti-Nek7 and anti-Ki-67 antibodies. **A.** The expression status of Nek7 in low-grade HCC tissue. **B.** An example of moderate-differentiated HCC with Nek7 expression. **C.** Nek7 expression in high-grade HCC tissue. **D.** Ki-67 expression status in low-grade HCC tissue. **E.** Example of moderate-differentiated HCC with Ki-67 expression. **F.** Ki-67 expression in high-grade HCC tissue.

Additionally, immunohistochemistry (IHC) staining of Nek7 also showed that all non-tumorous tissues expressed Nek7 at lower levels, while HCC tissues with different grades of tumorigenesis showed apparently higher levels of Nek7 expression (Figure 2A, 2B and 2C). The clinico-pathological features of HCC patients are reported in Table 1. The median and mean protein expression of Nek7 (82.0% and 81.9%, respectively) in HCC tissue was significantly higher compared to the median and mean in both normal (11.0% and 14.8%, respectively) and adjacent tissues (43.0% and 46.0%, respectively) (*p* < 0.001, Table 2).

**Table 1:** Characteristics of HCC patients with survival information (N = 120)

	Clinico-pathological features	Frequency N (%)
**Diagnosis**	hepatocellular carcinoma	120(100.0)
**Sex**	Female	42(35.0)
	Male	78(65.0)
**Age(year)**	<50	34(28.3)
	≥50	86(71.7)
**Tumor numbers**	Solitary	47(58.3)
	Multiple	73(41.7)
**Tumor diameter(cm)**	>5	74(61.7)
	≤5	46(38.3)
**Alpha fetoprotein (ug/l)**	<400	36(30.0)
	≥400	84(70.0)
**Tumor grade**	G1	31(25.8)
	G2	56(46.7)
	G3	33(27.5)
**TNM stage**	I and II	63(52.5)
	III and IV	57(47.5)
**Survival time (months)**	Median (range)	31(2-96)

**Table 2:** Differences in Nek7 expression between normal tissue, adjacent and HCC

	Normal (N=24)	Adjacent (N=76)	HCC (N=204)	*P-value*
**Mean ± SD**	14.8 ± 6.7	46.0 ± 9.3	81.9 ± 14.6	<0.001
**Median (min–max)**	11.0 (0–30)	43.0 (20–60)	82.0 (40–90)	

Next, we examined the correlation between the expression level of Nek7 and clinico-pathological features of HCC patients. High expression level of Nek7 was significantly correlated with tumor numbers, tumor diameter, adjacent organs invasion, tumor grade and TNM stage (Table 3). On the other hand, there was no correlation between Nek7 expression and age, portal vein invasion and Child-Pugh. Notably, there was no significant difference in the mean expression level of Nek7 between HCC patients with high and low AFP levels (< 200 vs. ≥200 ng/mL).

**Table 3:** Correlation between the status of Nek7 staining and clinico-pathological features in HCC patients

Clinico-pathological feature		number	Mean expression of Nek7%	T-value	*P*-value
**Age(year)**	**>50**	192	86.6%	0.659	0.534
	**≤50**	12	82.8%		
**HBV infection**	**Present**	154	81.8%	0.714	0.542
	**Absent**	50	83.5%		
**HCV infection**	**Present**	18	80.7%	0.662	0.501
	**Absent**	186	81.5%		
**Tumor numbers**	**Solitary**	106	71.7%	4.463	0.011
	**Multiple**	98	87.4%		
**Tumor diameter(cm)**	**>5**	97	84.8%	3.246	0.029
	**≤5**	107	59.0%		
**Adjacent organs invasion**	**Present**	105	83.6%	1.279	0.035
	**Absent**	99	67.8%		
**Portal vein invasion**	**Present**	74	80.2%	0.465	0.613
	**Absent**	130	79.7%		
**AFP (ng/mL)**	**<200**	69	86.4%	0.768	0.552
	**≥200**	135	84.9%		
**Child-Pugh**	**A**	109	79.9%	0.691	0.602
	**B**	75	80.4%		
	**C**	20	81.6%		
**TNM stage**	**I, II**	154	72.7%	1.482	0.026
	**III, IV**	50	87.5%		
**Tumor grade**	**G1, G2**	172	71.0%	1.506	0.019
	**G3**	32	84.6%		

### Correlation between expression levels of Nek7 and Ki-67

Since the Ki-67 protein is a well-established biomarker for cell proliferation [13], we sought to examine the correlation between its expression pattern and Nek7. Similar to what was observed for Nek7, IHC showed a differential staining intensity of Ki-67 among HCC tissues with different grades of tumorigenesis (Figure 2D, 2E and 2F). Statistically, the mean expression of Ki-67 protein was significantly higher in HCC tissue than that in normal and adjacent tissues (79%, 17% and 42%, respectively; *P* < 0.001). More importantly, Table 4 showed the correlation between Nek7 and Ki-67 expression, the results indicated that Nek7 played an important role in HCC proliferation.

**Table 4:** Association of Nek7 and Ki-67 expression

Nek7	Ki-67 Freq (%)		*p*-value
	Negative	Positive	
**Negative**	75(74)	33(16)	
**Positive**	26(26)	170(84)	<0.001
**Total number of cases**	101	203	

### Survival rate of HCC patients based on expression pattern of Nek7 and Ki-67

**Figure 3:**
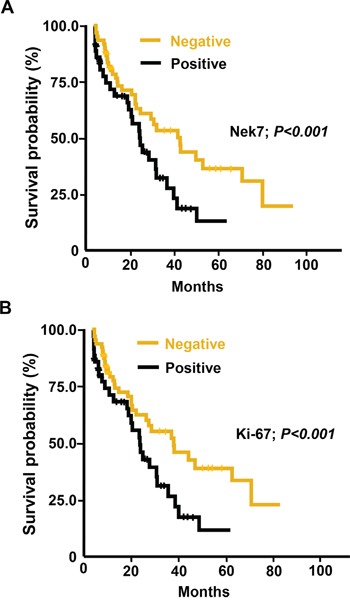
Kaplan-Meier analysis of overall survival in HCC patients according to Nek7 and Ki-67 expression **A.** Higher Nek7 expression was closely correlated with poor overall survival (*p* < 0.001). **B.** The HCC group with Ki-67 higher expression indicated poorer overall survival (*p* < 0.001).

To investigate the impact of Nek7 expression on the HCC outcome, Kaplan-Meier analysis was performed to compare the survival rate between HCC patients who were negative to Nek7 and HCC patients who were positive to Nek7. This analysis revealed that the 5-years survival rate was significantly higher in Nek7-negative patients than Nek7-postive patients (42% vs. 16%; *P* < 0.001) (Figure 3A). Similarly, the 5-years survival rate was significantly higher in Ki-67-negative patients than Ki-67-postive patients (42% vs. 13%; *P* < 0.001) (Figure 3B).

### Down-regulation of Nek7 expression by lenti-shRNAs inhibit HCC cell proliferation

**Figure 4:**
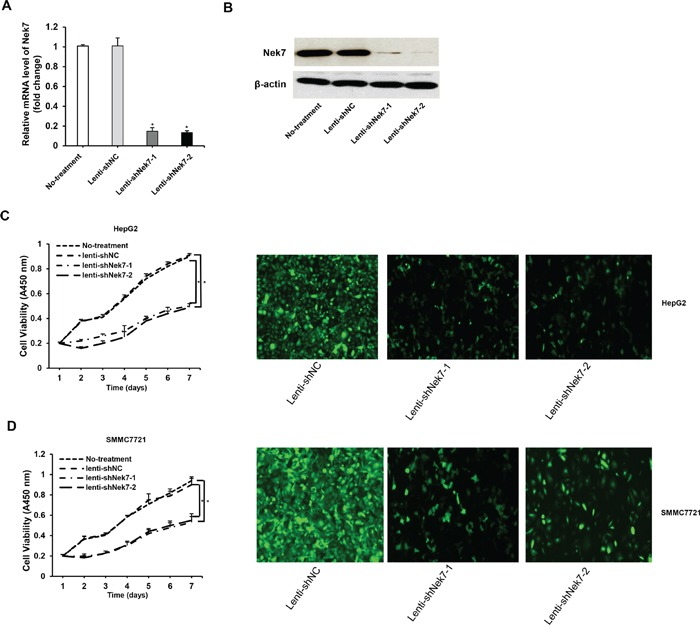
Lenti-shRNAs mediated down-regulation of Nek7 inhibited HCC cell growth HepG2 cells were infected with lenti-shNek7-1, -2 and negative control lenti-shNC. At 48 h post-transfection, the mRNA and protein levels of Nek7 expression were determined using RT-PCR **A.** and Western blot **B.** analyses. β-actin was used as internal control for Western blot and RT-PCR. HepG2 **C.** and SMMC7721 **D.** cells were infected with lenti-shNek7-1, -2 and negative control lenti-shNC, respectively. Cell viability was determined using the CCK-8 assay 7 days after infection. Nek7 expression was detected in HCC cells via immunohistochemical staining with anti-Nek7 antibody under a confocal laser scanning microscope. Experiments were repeated three times. (*p < 0.05).

In attempt to elucidate the link between Nek7 and HCC, we investigate the effect of Nek7 down-regulation on proliferation of HCC cell lines. For this end, HepG2 cells were infected with either control lenti-shNC or a Nek-7 specific lenti-shRNA (lenti-shNek7-1 and -2.) Following infection, real-time PCR and western blot assays were performed to determine Nek7 mRNA and protein expression levels. As shown in Figure 4A, 4B either lenti-shNek7-1 or lenti-shNek7-2 effectively inhibited both Nek7 gene transcription (up to 80% down-regulation compared to cells transfected with control lenti-shNC) and protein expression (up to 90% knock-down compared to the control).

Next, we examined of the impact of Nek7 down-regulation on the growth rate of HCC cells. To address this, HepG2 and SMMC7721 cells were seeded in 96-well plates, then infected with either Nek7-shRNA lentivirus or shRNA-NC lentivirus. The CCK-8 method was used to analyze cell viability 7 days post infection. The result showed that the viability of HepG2 and SMMC7721 cells infected with Nek7 specific lenti-shRNA was significantly inhibited compared to the control cells, indicating that down-regulation of Nek7 negatively regulates the cell growth rate (Figure 4C, 4D).

### Knockdown of Nek7 expression inhibited SMMC7721 xenograft tumors growth

**Figure 5:**
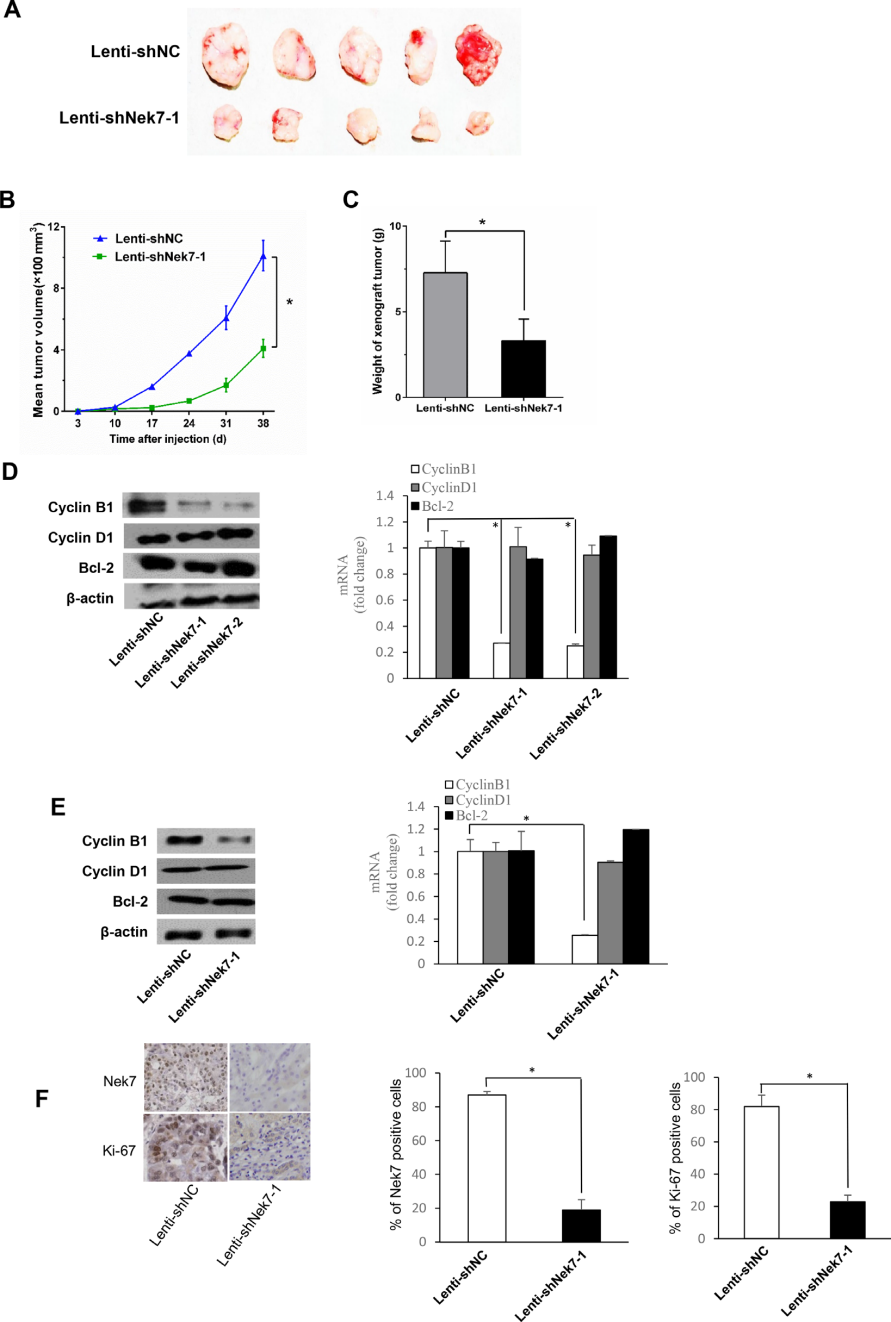
Nek7 gene silencing suppressed growth of SMMC7721 xenograft tumors **A.** Tumors isolated from mice of different groups are shown. **B.** Growth curves of xenograft tumors from the experiments with BALB/c nude mice. Changes in tumor volumes measured at the indicated days are shown. **C.** Therapeutic lenti-shNek7-1 reduced the tumor weight. Real-time PCR and Western blot assays to determine the mRNA and protein levels of cell growth associated genes, Bcl-2, cyclinD1 and cyclinB1 in HCC cells **D.** and dissected tumors **E, F.** Immunohistochemical staining was used to detect the Nek7 and Ki-67 expression pattern in xenograft tumors from different groups. β-actin was used as internal control for Western blot and Real-time PCR. Experiments were repeated three times. (*p < 0.05).

To further validate the *in vitro* results, a two groups of xenograft mice were generated: (i) control-group which included BALB/c nude mice inoculated with SMMC7721 cells infected with lenti-shNC and (ii) treated-group which included BALB/c nude mice inoculated with cells infected with lenti-shNek7-1. The xenograft tumor taken rate is 100% in nude mice. Interestingly, 5 weeks post inoculation, the size and the weight of tumors obtained from mice of treated-group were significantly smaller than that from mice of control-group (Figure 5A, 5B, 5C). These results clearly indicated that Nek7 is critical for HCC cell proliferation and tumorigenicity.

In addition, we examined the expression of apoptotic and proliferation genes including Bcl-2, cyclinD1 and cyclinB1 in HepG2 and SMMC7721 cells and tumor of xenograft mice. The expression levels of CyclinB1 mRNA and protein were decreased in either HCC cells transfected with Nek7-specific lenti-shRNA (Figure 5D) or in tumors obtained from treated-group of xenograft mice (Figure 5E). On the other hand, there is no impact of Nek7 down-regulation on Bcl-2, cyclinD1. Moreover, the isolated tumor tissues were subjected to IHC staining for Nek7 and Ki-67. The results showed that the Nek7 and Ki-67 expression significantly decreased in treated-group compared to control-group (Figure 5F).

## DISCUSSION

Hepatocellular carcinoma (HCC) is a primary cancer of the liver, which causes approximately 600,000 deaths globally each year [1]. The prognosis of most HCC patients is typically poor since more than 80% HCC is diagnosed at an advanced stage with high resistance to chemotherapy and radiotherapy [19]. Although many gene signatures and signaling pathways have been identified in liver tumors that can be used for early detection, diagnosis and gene target therapy, additional novel molecular markers are still potentially needed [4, 20-21]. In this context, the deregulation of cell cycle and checkpoint machinery can lead to cancer, which also presents a highly attractive therapeutic strategy [22]. Nek7 is one of the members of the NIMA related protein kinase (Nek) family involved in cell cycle control [11]. Recently, many studies provide evidences that Nek kinase has close relationship with cancer progression through regulating cell cycle and spindle checkpoint [23]. Notably, it has been reported that Nek7 is overexpressed in gallbladder cancer tissues, however, until now there is no data about the expression pattern and biological role of Nek7 in HCC [12].

In the present study, we examined Nek7 expression in HCC cell lines and cancer tissues. Compared with the normal liver cell line LO2 and normal liver tissues, Nek7 highly overexpressed in HCC cell lines and HCC tissues suggesting that expression pattern of Nek7 correlates with HCC progression. Moreover, Nek7 expression associated with tumor numbers, tumor diameter, adjacent organs invasion, tumor grade and TNM stage in HCC patients. Also, we found a close association between Nek7 and Ki-67 expression patterns. This indicates Nek7 has an important role in HCC cell proliferation. More importantly, high expression of Nek7 predicted poor survival rate compare with the low-expression groups. Therefore, obtained clinical and experimental results emphasize the involvement of Nek7 in HCC progression.

To explore the biological role of Nek7 in HCC cells, we found that Nek7 silencing significantly inhibited cell growth of HCC cell lines. Furthermore, *in vivo* experiment demonstrated that the tumor growth in xenograft mice with Nek7/knock-down SMMC7721 cells was obviously suppressed. Mechanistic analysis demonstrated that down-regulated Nek7 decreased cyclinB1 expression in HCC cells and xenograft models. Since cyclin B1 plays an important role in initiating the progression from G2 phase to mitosis by regulating Cyclin-dependent kinase 1 (Cdk1) [24-25], thus it is quite possible that the knock-down of Nek7 expression suppress HCC cell proliferation and tumorigenicity by regulating cyclin B1 expression. Future detailed mechanistic studies are needed to further explore this issue.

In summary, this study demonstrates for the first time that Nek7 is significantly overexpressed in HCC cell lines and tissues, and its expression pattern correlated with clinico-pathological features of HCC patients. More importantly, expression pattern of Nek7 could be used as a biomarker for poor survival. In addition, Nek7 might involve in HCC progression by regulating cyclin B1 expression. Taken together, this study presents Nek7 as a novel molecular target for HCC therapy.

## MATERIALS AND METHODS

### Patients' specimens

120 HCC samples, 50 corresponding adjacent tissues and 10 normal liver tissues collected from patients with HCC who underwent complete surgical resection at the First Affiliated Hospital of Xi'an Jiaotong University (Xi'an, China) from January 2001 to January 2010. All the patients were confirmed hepatocellular carcinoma by pathological examination with no prior chemotherapy or transhepatic arterial embolization history. Patients were followed-up for a period of 5 years. A tissue microarrays (TMA) (Biomax US) was used to analyze the Nek7 expression in 84 HCC samples, 26 adjacent tissues and 14 normal liver tissues. All patient samples were immediately frozen and stored in liquid nitrogen until they used in further investigations. Informed consent was obtained from all patients, and the study protocol was approved by our institutional review board of the First Affiliated Hospital, School of Medicine, Xi'an Jiaotong University. The characteristics of the HCC patients were summarized in Table 1.

### Cell lines

Human HCC cell lines including HepG2, Huh7, Hep3B and SMMC7721, and a human normal hepatic cell line LO2 and human embryonic kidney 293T were purchased from the American Type Culture Collection (ATCC). HepG2, SMMC7721 and LO2 were cultured in RPMI 1640 medium with 10% superior placental bovine serum (Sijiqing, HangZhou, China). Huh7, Hep3B and 293T cells were maintained in Dulbeccos' Modified Eagle's Medium (DMEM; Hyclone, NJ, USA) supplemented with 10% FBS, 100 units/ml of penicillin and 100 μg/ml streptomycin.

### RNA isolation and determination of gene expression using semi-quantitative RT-PCR and quantitative real-time PCR

Total RNA was purified from hepatic tissue and cell lines using RNAfast200 Total RNA Extract Kit (Fastgene, ShangHai, China). The cDNA was prepared by RevertAid™ First Strand cDNA Synthesis Kit (Fermentas, MBI, Lithuania). Resulted cDNA was subjected to conventional PCR using PrimeScript™ One Step RT-PCR Kit Ver.2 (Takara, DaLian, China) for semi-quantitative assay and to real-time reverse transcription-polymerase chain reaction (RT-PCR) was performed using an RT-PCR kit according to the protocols recommended by the manufacturer. A SYBR green-based RT-PCR assay was used to determine the mRNA level of Nek7 in HCC and matched normal adjacent liver tissues using the sequence detection system (BIO-RAD, California, USA). The relative Nek7 levels in each sample were normalized to β-actin cDNA and calculated using *ΔΔ*CT methods. Primers used for amplification were as follows: β-actin, forward primer, 5′-AAGGAAGGCTGGAAGAGTGC-3′, reverse primer, 5′-CTGGGACGACATGGAGAAAA-3′; Nek7, forward primer, 5′-CACCTGTTCCTC AGTTCCAAC-3′, reverse primer, 5′-CTCCATCCAAGAGACAGGCTG-3′.

### Immunohistochemical analysis and evaluation

All paraffin-embedded HCC tissues and surrounding non-tumor tissues were cut into 4-μm-thick serial sections. Slides were deparaffinized in xylene, rehydrated in graded alcohol, immersed in 3% hydrogen peroxide for 10 mins to block endogenous peroxidase activity. After heat-induced epitope retrieval, the slides were incubated with a primary Nek7 antibody (monoclonal mouse; 1:200; Santa Cruz, CA, USA) for 1 hour at room temperature. Subsequently, slides were incubated with HRP-labeled secondary antibody (1:100) (Boster corp, WuHan, China) at 37°C for 30 min. DAB (3, 3-diaminobenzidine) was used as a chromagen, and sections were counterstained with hematoxylin, dehydrated and mounted. The tissue microarray slides were performed based on the manufacturer's standard protocols. A negative control was obtained by replacing the primary antibody with a normal rabbit or mouse IgG. For Ki-67 staining a rabbit monoclonal antibody (Boster corp, WuHan, China) at a dilution 1:100 was used.

Nuclear Nek7 and Ki-67 staining were scored by two independent observers blinded for clinical parameters. Slides were screened semi-quantitatively for the percentage of positivity and intensity of the signal [26]. We scored the staining intensity as follows: no staining of cells in any microscopic field, 0; < 30% of tissue stained positive, 1+; 30% ∼ 60% of tissue stained positive, 2+; > 60% of tissue stained positive, 3+.

### Western blot analysis

The hepatic tissue and cell lines were ground and lysed in RIPA buffer on ice and then subjected to Western blot analysis. Equal amounts of proteins were loaded onto SDS-polyacrylamide gels, and the resulting bands were transferred to a polyvinylidene difluoride membranes (Millipore, MA, USA). The membrane was blocked and then incubated with primary and secondary antibodies. The primary antibodies used were raised against Nek7 (Santa Cruz, CA), Bcl-2 (Boster corp, WuHan), Cyclin B1 and Cyclin D1 (Cell Signaling Technology, MA). Signals were detected on X-ray film using an ECL detection system (Pierce, Rockford, IL, USA).

### Construction and synthesis of shRNA fragments

Two shRNAs against Nek7 were designed using the Whitehead Institute Web Server (http://jura.wi.mit.edu/bioc/siRNAext/) and were chemically synthesized (Shanghai GenePharma Co.). These two Nek7 shRNAs were utilized to target different regions of Nek7 transcript. The shNek7-1, was designed to be homologous to nucleotides 328-350 of the human Nek7. The sequence of this duplex is: 5′-GGAUGAGCAAUCACAAGGA-3′ (sense), 5′-UCCUUGUGAUUGCUCAUCC-3′ (antisense). The shNek7-2 targeted Nek7 mRNA at nucleotides 718–740. The sequence of shNek7-2 is: 5′-GCUAAUUCCUGAAAGAACU-3′ (sense), and 5′-AGUUCUUUCAGGAAUUAGC-3′ (antisense). The sequence of control shRNA (shNC) is: 5′-CGUCAGAGUAUACUAAUAU-3′ (sense) and 5′-AUAUUAGUAUACUCUGACG-3′ (antisense).

### Clone shRNA oligos into lentivirus vector

These shRNA oligos were cloned into lentivirus vector pLKO.1 followed the instruction provided by Addgene (Boston, MA). Lentiviral particles were generated by calcium phosphate-mediated co-transfection of HEK-293T cells with shNek7-1/pLKO.1, shNek7-2/pLKO.1, or shNC/pLKO.1 together with pCMV-dR8.2 dvpr (Packaging plasmid) and pCMV-VSVG (Envelope plasmid). Lenti-shNek7-1, Lenti-shNek7-2, or Lenti-shNC was harvested 48 hours after transfection. The viral supernatant was filtered with a 0.45 μm filter and concentrated by ultracentrifugation. The final titer of recombinant lentivirus was 2.0×10^9^ infectious unit/ml, and stored at −70°C. Knockdown efficiency was determined by real-time PCR and western blot.

### CCK-8 assay for cell growth

HepG2 and SMMC7721 cells were seeded at 5×10^3^ per well in 96-well flat-bottom plates and incubated in humidified incubators at 37°C with 5% CO_2_. The cells were grown to 30%∼50% confluence and then transfected with lenti-shNC or lenti-shNek7-1, -2 using the polybrene (6μg/ml) transfection reagent (Santa Cruz, CA). The cells were then sub-cultured at 24-hour intervals for 7 days. The cell viability was measured using CCK-8 (Dojindo, Japan) according to the manufacturer instructions. The absorbance was measured at 450 nm by a microplate reader (Bio-Rad, Hercules, CA, USA) to assess the cell viability. All experiments were independently repeated at least 3 times.

### Tumor xenograft experiments

After infection with lenti-shNC or lenti-shNek7-1, SMMC7721 cells were trypsinized and counted. Four-week-old BALB/c nude mice (n=5 for each group) were injected subcutaneously in the right flank with 5 ×10^6^ SMMC7721 cells suspended in 200 μl of phosphate-buffered saline for experimental tumorigenicity assays. Mice were weighed and the tumor width (W) and length (L) were measured for approximately 5 weeks, and calculated using the formula: (length ×width^2^)/2. Tumors were excised from the animals, and then frozen in liquid nitrogen all animal procedures were conducted in accordance with the policies and regulations of Xi'an Jiaotong University Institutional Animal Care and Use Committee (Xi'an, China).

### Statistical analysis

Differences in Nek7 and Ki-67 expression in different sample groups were investigated using the non-parametric Wilcoxon Rank Sum Test. Chi-square tests were used to study the relationship between Nek7 and Ki-67 expression and histological subgroups. The Fisher's exact test was employed to evaluate the relationship between Nek7 expression and clinico-pathological variables. The survival probability analysis was carried out by the Kaplan-Meier and the difference between groups was tested by log-rank test. Differences in the mean and median Nek7 expression between normal tissue, adjacent and HCC were analyzed by one-way ANOVA. The *P* < 0.05 was considered to be significant. All analyses were performed using SPSS version 16.0 (SPSS, Inc., Chicago, IL).
